# Apigenin is a promising molecule for treatment of visceral leishmaniasis

**DOI:** 10.3389/fcimb.2023.1066407

**Published:** 2023-04-05

**Authors:** Yago S. S. Emiliano, Elmo E. Almeida-Amaral

**Affiliations:** Laboratório de Bioquímica de Tripanosomatideos, Instituto Oswaldo Cruz (IOC), Fundação Oswaldo Cruz – FIOCRUZ, Rio de Janeiro, RJ, Brazil

**Keywords:** natural products, oral treatment, flavonoid, short-term, long-term, *in vivo*, *in vitro*, leishmaniasis chemotherapy

## Abstract

Current treatment for visceral leishmaniasis is based on drugs such as pentavalent antimony and amphotericin B. However, this treatment remains mostly ineffective and expensive, resulting in several side effects and generating resistance. Apigenin, a flavonoid present in fruits and vegetables, has demonstrated several biological functions. In the present study, we observed a concentration-dependent inhibition of the *L. infantum* promastigote in the presence of apigenin, exhibiting an IC_50_ value of 29.9 µM. Its effect was also evaluated in *L. infantum*-infected murine peritoneal macrophages, presenting an C_50_ value against intracellular amastigotes of 2.3 µM and a selectivity index of 34.3. In a murine model of visceral leishmaniasis, the *in vivo* effect of apigenin was measured using short-term and long-term treatment schemes. Treatment with apigenin demonstrated 99.7% and 94% reductions in the liver parasite load in the short-term and long-term treatment schemes, respectively. Furthermore, no alterations in serological and hematological parameters were observed. Taken together, these results suggest that apigenin is a potential candidate for visceral leishmaniasis chemotherapy by oral administration.

## Introduction

Leishmaniasis is a neglected tropical disease that is endemic in 98 countries and present in 200 territories, and 12 million people are affected worldwide. Visceral leishmaniasis is a form of this disease with a 95% fatality rate in untreated cases, and it is estimated that 30,000 new cases occur annually (Ruiz-Postigo et al., 2021). This disease has a high prevalence in Brazil, where 96% of cases are reported (Pan American Health Organization, 2018).

In endemic areas of visceral leishmaniasis, a rise in Leishmania–HIV coinfection has been observed. Visceral leishmaniasis accelerates HIV replication and progression, and HIV can influence the disease’s epidemiology, clinical manifestations, and course. Additionally, *Leishmania*–HIV coinfected patients are more difficult to treat, and they do not respond well to standard treatments, facing more frequent and more severe side effects and higher risks of disease recurrence and death (Alvar et al., 2008; van Griensven and Diro, 2012; DNDi, 2022).

Current treatment for visceral leishmaniasis is based on drugs such as pentavalent antimonials, which have been used as first-line agents for many years, and amphotericin B deoxycholate and lipid formulation amphotericin B (Uliana et al., 2017). These drugs are administered intramuscularly, leading to long-established administration, high cost, and high toxicity. Additionally, pentavalent antimonials are becoming increasingly ineffective due to resistance, and liposomal amphotericin B is a high-cost formulation, making this drug prohibitive in several endemic regions (Uliana et al., 2017; Zulfiqar et al., 2017). Miltefosine has emerged as an alternative treatment for visceral leishmaniasis; however, it is expensive and teratogenic, and its use is not licensed worldwide (Rijal et al., 2013). Therefore, it is necessary to search for new alternatives for the treatment of visceral leishmaniasis. In this context, several natural products have been demonstrated to have antileishmanial activities (Gervazoni et al., 2020).

We had previously demonstrated that apigenin, a flavonoid present in common fruits and vegetables, such as parsley, lemons and berries, has antiparasitic activity *in vitroin vitro* against *Leishmania amazonensis*-infected macrophages and can be used as an oral treatment in an experimental model of cutaneous leishmaniasis caused by *L. amazonensis* (Fonseca-Silva et al., 2016; Emiliano and Almeida-Amaral, 2018). In the present study, we investigated the *in vitro* activity of apigenin against promastigote and intracellular amastigotes of *Leishmania infantum*, the etiological agent of visceral leishmaniasis in the New World, and its *in vivo* activity in an experimental model of visceral leishmaniasis caused by *L. infantum*.

## Materials and methods

### Test compound and reagent

Apigenin (molecular formula: C15H10O5; molecular weight: 270.24 g/mol; purity ≥95%; lot WE445301/1) and other reagents were obtained from Merck KGaA. Apigenin was diluted in dimethyl sulfoxide (DMSO) such that the solvent concentration did not exceed 0.2% in the final solution and added to the culture medium. Endotoxin-free sterile disposables were used in all experiments. The chemical structure of apigenin is presented in **Supplementary Figure 1**.

### Ethics statement

This study was performed in strict accordance with the recommendations of the Guide for the Care and Use of Laboratory Animals of the Brazilian National Council of Animal Experimentation (CONCEA). The protocol was approved by the Committee on the Ethics of Animal Experiments of the Instituto Oswaldo Cruz (CEUA-IOC, License Number: L-11/2017 A2).

### Parasites and mice

In this study, we used a strain of *L. infantum* (MHOM/MA/67/ITMAP263). *L. infantum* promastigotes were cultivated at 26°C in Schneider’s *Drosophila* medium (pH 6.9) supplemented with 20% fetal bovine serum (v/v), 100 μg/mL streptomycin, and 100 U/mL penicillin. Parasite maintenance was promoted by culture passage every 3 days. Female BALB/c mice (8-10 weeks; provided by the Instituto Ciencias e Tecnologia em Biomodelos, ICTB/FIOCRUZ) were used in this study. All animals were bred and maintained at the Instituto Oswaldo Cruz according to the Guide for the Care and Use of Laboratory Animals of the Brazilian National Council of Animal Experimentation (CONCEA).

### Promastigote proliferation assay

*L. infantum* promastigotes (1.0 x 10^6^ cell/mL) were incubated in the absence (DMSO 0.2%) or presence of different concentrations of apigenin (12 µM, 24 µM, 36 µM, 48 µM, 60 µM, 72 µM, 80 µM and 96 µM) for 72 h. Cellular proliferation was determined by the Alamar Blue assay. Fluorescence was monitored at excitation and emission wavelengths of 560 and 590 nm, respectively, using a spectrofluorometer. The 50% inhibitory concentration (IC_50_) was determined by logarithmic regression analysis using GraphPad Prism 7 (GraphPad Software, La Jolla, CA, USA). The experiments were performed in triplicate.

### Leishmania-macrophage interaction assay

Peritoneal macrophages were collected from BALB/c mice in RPMI 1640 medium and plated at 2.0 x 10^6^ macrophages/mL (0.4 mL/well) onto Lab-Tek eight-chamber slides for 1 h at 37°C in an atmosphere of 5% CO_2_. Stationary-phase *L. infantum* promastigotes were washed with PBS, counted in a Neubauer chamber, and added to macrophages at a multiplicity of infection (MOI) of 5:1 for 5 h at 37°C in an atmosphere of 5% CO_2_. Then, 2% heat-inactivated horse serum (v/v) was added for 18 hours (Inacio et al., 2019). After this, successive washes with RPMI 1640 medium were performed to remove free parasites. Then, *L. infantum*-infected macrophages were incubated in the absence (DMSO 0.2%) or presence of different concentrations of apigenin (3 µM, 6 µM, 12 µM and 24 µM) for 72 hours and stained using Instant Prov (Newprov, Curitiba/Brazil). The percentage of infected macrophages was determined by light microscopy, and at least 200 cells on each coverslip were randomly counted in duplicate. The results are expressed as the infection index (% infected macrophages x number of amastigotes/total number of macrophages). The IC_50_ was determined by logarithmic regression analysis using GraphPad Prism 7. The selectivity index (SI) was obtained as murine peritoneal macrophage CC_50_/intracellular amastigote IC_50_. The macrophage CC_50_ for apigenin is 78.7 µM (Fonseca-Silva et al., 2016).

### *In vivo* infection using a murine model of visceral leishmaniasis and parasite load quantification

BALB/c mice (five animals per group) were maintained under specific pathogen-free conditions. The animals were infected *via* the peritoneum with 100 µL of stationary-phase *L. infantum* promastigotes at a concentration of 1.0 x 10^8^ cells/ml as described (Cunha-Junior et al., 2016). At 7 days post-infection, the animals were separated into three different groups (control group, apigenin-treated group, and positive control group). The positive control group was treated using meglumine antimoniate. At the end of the treatment, mice were euthanized, and the livers were removed, weighed, and macerated in Schneider’s medium with 20% FBS for analysis of the parasite load by limiting dilution assay (LDA). The number of viable parasites in each liver was estimated from the highest dilution at which promastigotes could be grown after 7 days of incubation at 26°C (Inacio et al., 2019).

### Short-term therapeutic scheme

In the short-term therapeutic treatment scheme, the infected mice, divided into three groups, were treated with vehicle (DMSO; 0.2% v/v), which was incorporated in an oral suspension and administered orally by gavage twice per day (control group); apigenin (1 mg/kg diluted in DMSO 0.2% v/v), which was incorporated in an oral suspension and administered orally by gavage twice per day, reaching a maximum daily dose of 2 mg/kg/day (apigenin-treated group); and meglumine antimoniate, which was administered intramuscularly once per day at a dose of 200 mg Sb^5+^/kg/day (positive control group). These treatments started 7 days post-infection and lasted 5 days. At the end of the experiment (Day 14), the animals were euthanized, and the liver was obtained for analysis of the parasite load by limiting dilution assay (LDA) as described above.

### Long-term therapeutic scheme

In the long-term therapeutic treatment scheme, *L. infantum*-infected mice were divided into three groups and treated with vehicle (DMSO 0.2% v/v), which was incorporated in an oral suspension and administered orally by gavage twice per day (control group); apigenin (1 mg/kg diluted in DMSO 0.2% v/v), which was incorporated in an oral suspension and administered orally twice per day by gavage, reaching a maximum daily dose of 2 mg/kg/day (apigenin-treated group); and meglumine antimoniate, which was administered intramuscularly once per day at a dose of 100 mg Sb^5+^/kg/day (positive control group). The animals were treated for 5 consecutive days beginning 7 days post-infection. At 18 days after treatment (Day 30), mice were euthanized, and the livers were obtained for analysis of the parasite load by limiting dilution assay (LDA) as described above.

### Toxicology

Before euthanasia, BALB/c mice were anesthetized with a solution of ketamine (200 mg/kg) and xylazine (16 mg/kg) administered intraperitoneally. Blood was collected (1 mL) *via* cardiac puncture and distributed in EDTA-containing microtubes for hematological analysis or centrifuged to obtain serum. Both serum (toxicology markers) and total blood (hematological parameters) from the infected BALB/c mice treated as described above were measured by Technological Platforms Network -FIOCRUZ, Platforms of Clinical Analysis in Laboratory Animals - RPT12C.

### Statistical analysis

The data were analyzed using the Mann−Whitney test or one-way analysis of variance (ANOVA) followed by Tukey’s post-test in GraphPad Prism 7 (GraphPad Software). The results were considered significant at p ≤ 0.05. The data are expressed as the means ± standard errors.

## Results

**Figure 1:**
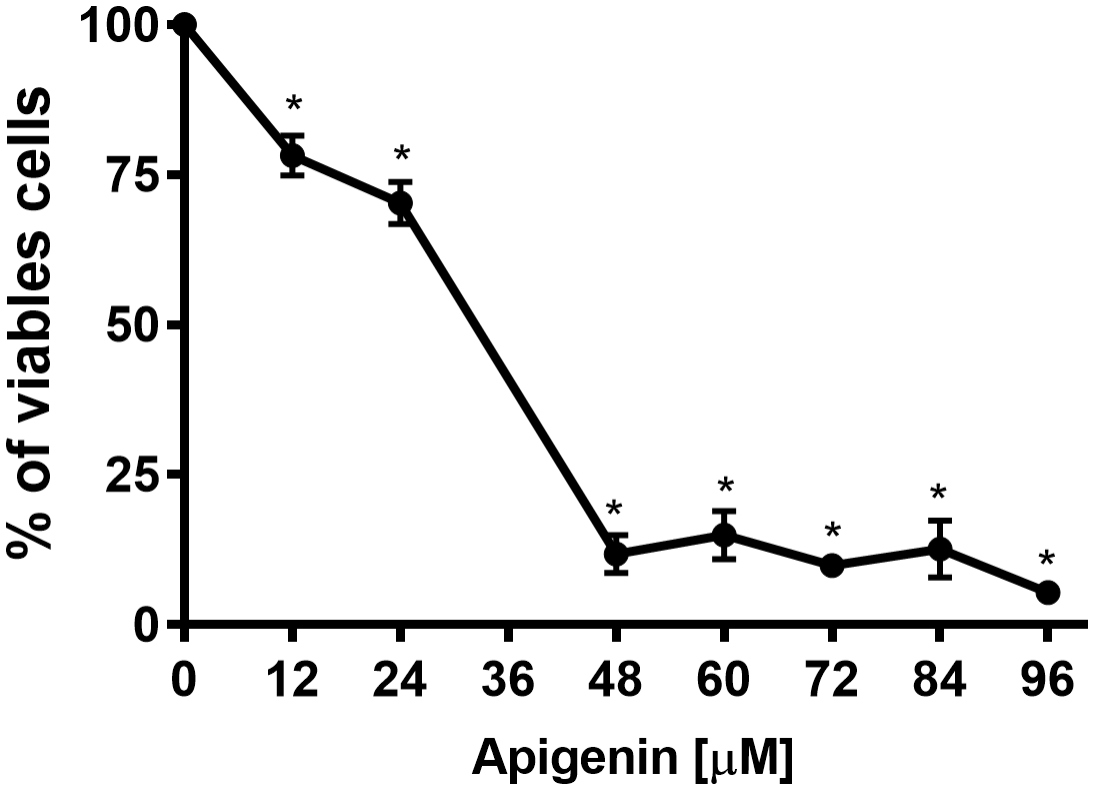
Effect of apigenin on *L. infantum* promastigotes. *L. infantum* promastigotes were incubated in Schneider’s *Drosophila* medium in the absence or presence of increasing concentrations of apigenin (12-96 µM) for 72 h. Cellular proliferation was evaluated using the Alamar Blue assay. The values are presented as the mean ± standard error of three different experiments. * indicates a significant difference relative to the control (p < 0.001).

To evaluate the *in vitro* effect of apigenin, promastigote forms and intracellular amastigote forms of *L. infantum* were used. Promastigotes were incubated in the absence or presence of different concentrations of apigenin (12-96 µM) for 72 h (**Figure 1**). Apigenin inhibited the cellular proliferation of *L. infantum* in a concentration-dependent manner, reaching 94.6% inhibition at the highest concentration (96 µM) with an IC_50_ value of 29.9 µM.

**Figure 2:**
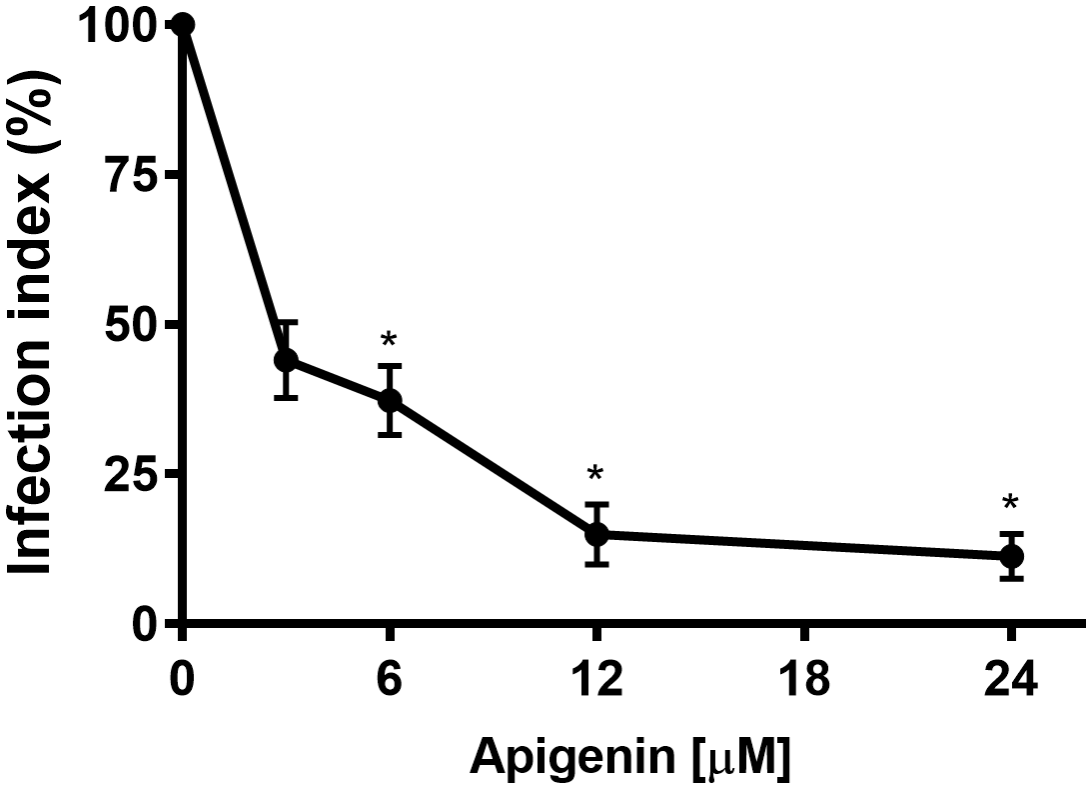
Effect of apigenin on *L. infantum*-infected macrophages. *L. infantum*-infected macrophages were incubated in the absence or presence of increasing concentrations of apigenin (3-24 µM) for 72 h. In control samples (absence of apigenin), a similar volume of vehicle (0.2% DMSO) was added to the cells. The infection index was determined using light microscopy to count at least 200 macrophages in each duplicated coverslip. The values presented refer to the mean ± standard error of three different experiments. * indicates a significant difference relative to the control (p < 0.05).

To determine the effect of apigenin on *L. infantum* intracellular amastigotes, peritoneal BALB/c mouse macrophages were infected with promastigotes of *L. infantum* for 5 h and then incubated with increasing concentrations of apigenin (3-24 μM) for 72 h. Apigenin was able to reduce the infection index in a concentration-dependent manner (**Figure 2**). The IC_50_ was 2.3 µM, reaching 88% inhibition at the highest concentration (24 µM). The reported CC_50_ value of apigenin is 78.7 µM (Fonseca-Silva et al., 2016), and the selectivity index (SI) was calculated to be 34.3.

According to the observed *in vitro* effect of apigenin, the efficacy of this molecule *in vivo* was evaluated using a murine model of visceral leishmaniasis using two therapeutic schemes. The first was short-term, where *L. infantum*-infected BALB/c mice were treated for 5 days beginning at 7 days post-infection and euthanized at 2 days post-treatment (Day 14). In the long-term therapeutic scheme, *L. infantum*-infected BALB/c mice were also treated for 5 days beginning at 7 days post-infection, but euthanization occurred at 18 days post-treatment (Day 30) (Le Fichoux et al., 1998).

**Figure 3:**
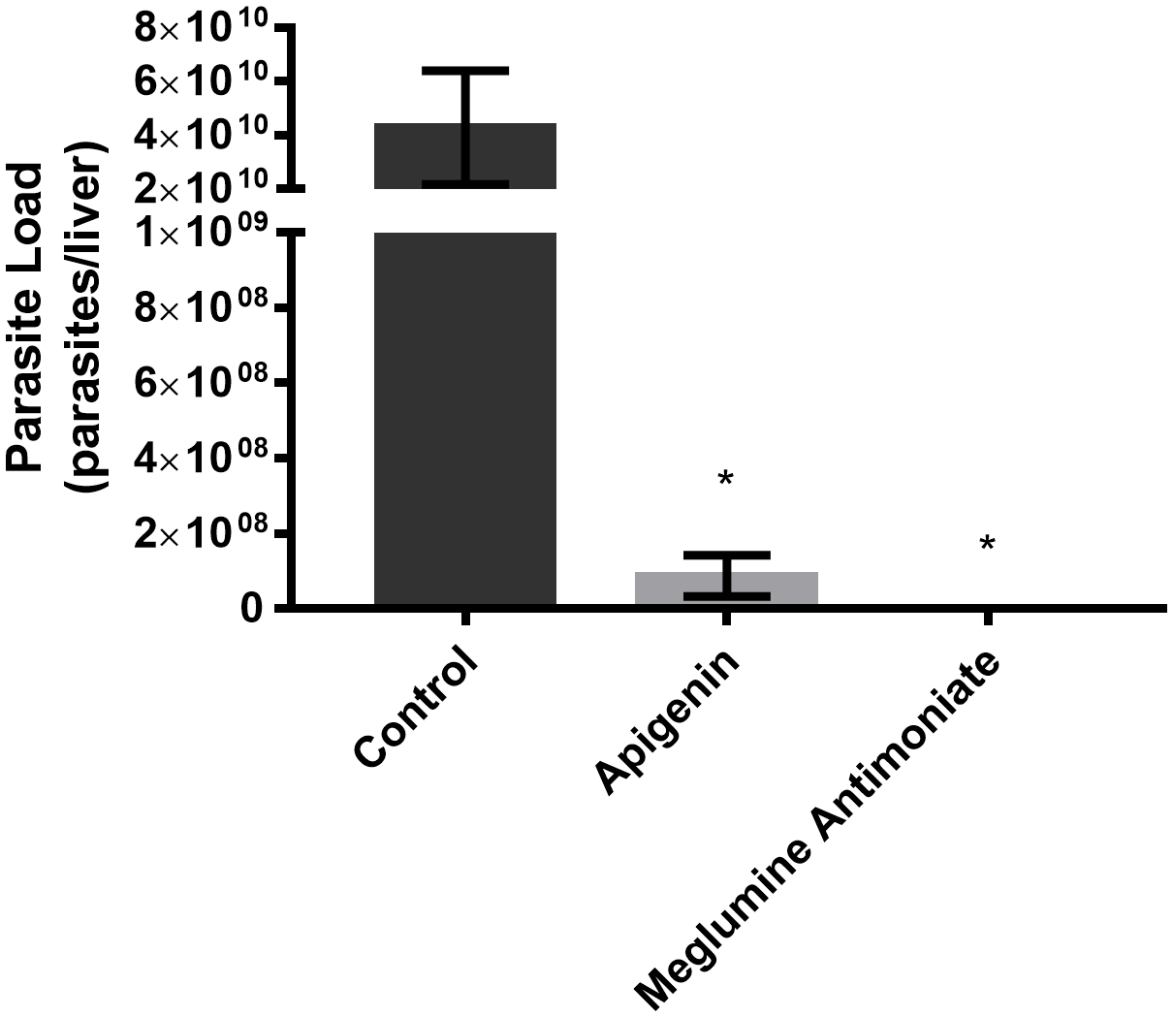
Short-term therapeutic efficacy of apigenin against *L. infantum*-infected BALB/c mice. Parasitic load of *L. infantum*-infected BALB/c mice (5 animals per group) untreated or treated with apigenin or meglumine antimoniate. Liver parasite load was estimated by a limiting dilution assay (LDA). These data represent one independent experiment with five mice per group (n = 5). * indicates a significant difference relative to the control group (p < 0.01).

As shown in **Figure 3**, during the short-term treatment, apigenin orally administered to *L. infantum*-infected BALB/c mice reduced the liver-parasite load, reaching 99.7% inhibition. However, we did not observe any significant differences between mice treated with apigenin and those treated with meglumine antimoniate.

Furthermore, serological toxicology parameters, such as total levels of albumin, creatinine kinase, urea, alkaline phosphatase, alanine aminotransferase (ALT), aspartate aminotransferase (AST), cholesterol, iron, calcium, sodium, and potassium (**Supplementary Table 1**), and several hematological parameters (**Supplementary Table 2**) were evaluated, and no significant alterations were observed, suggesting a lack of toxicity.

**Figure 4:**
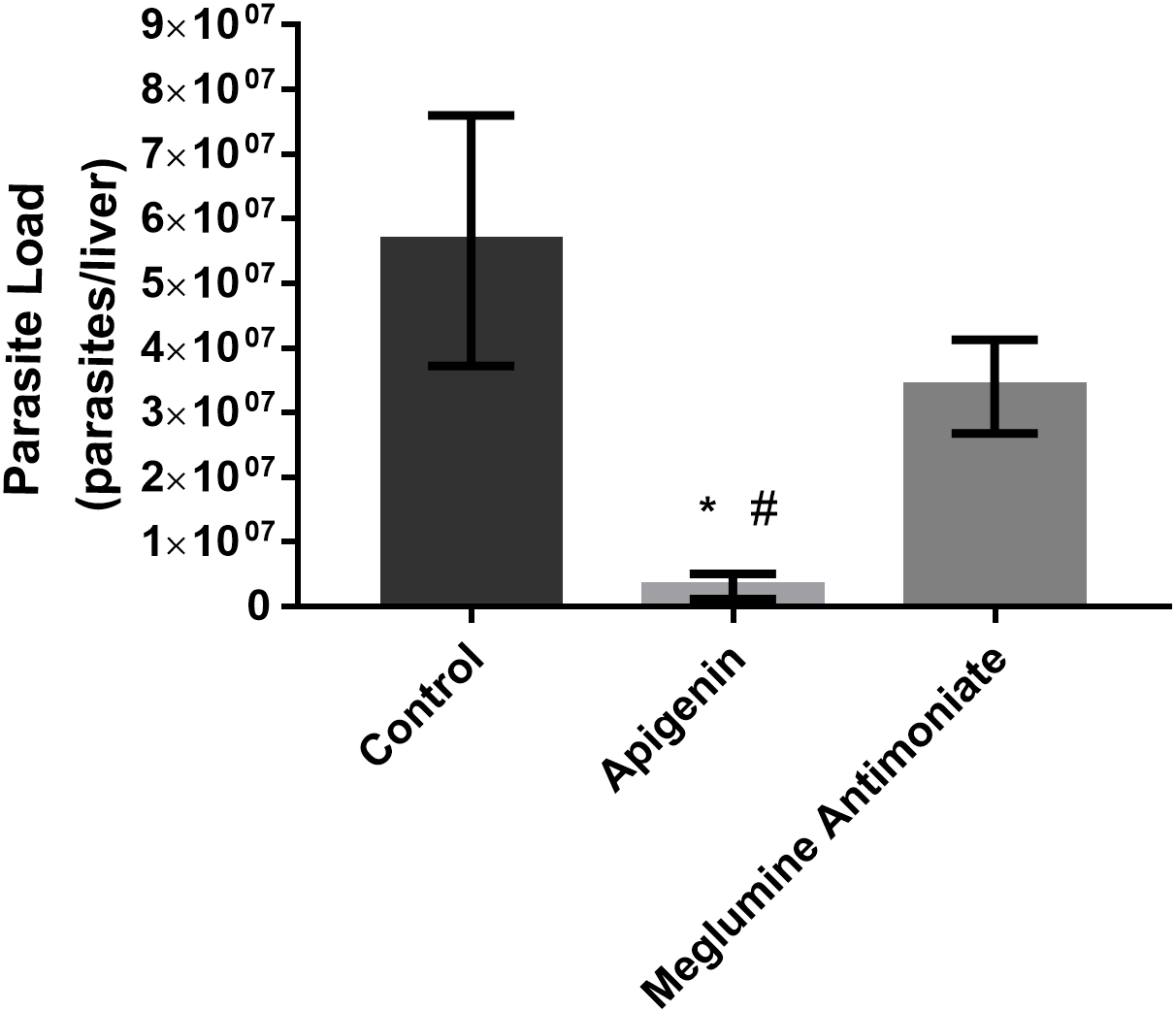
Long-term therapeutic efficacy of apigenin against *L. infantum*-infected BALB/c mice. Parasitic load of *L. infantum*-infected BALB/c mice (five animals per group) untreated or treated with apigenin or meglumine antimoniate. The liver parasite load was estimated by a limiting dilution assay (LDA). These data represent one independent experiment with five mice per group (n = 5). * indicates a significant difference relative to the control group (p < 0.01). # indicates a significant difference relative to the meglumine antimoniate group (positive control group) (p < 0.01).

In the long-term therapeutic scheme (**Figure 4**), oral administration of apigenin to *L. infantum*-infected BALB/c mice resulted in a 94% reduction in the liver parasite load. Furthermore, differences between the infected mice treated with apigenin and meglumine antimoniate were observed in terms of parasite load, and meglumine antimoniate reduced the liver parasite load by only 55%.

## Discussion

Current visceral leishmaniasis chemotherapy has presented many problems, such as several collateral effects, increased ineffectiveness due to resistance, and high cost making these drugs unaffordable in several endemic regions. Therefore, more research is necessary to improve this drug arsenal, giving priority to compounds with greater efficacy, reduced toxicity, and better affordability. Natural products have become an interesting alternative, and flavonoids have been studied as promising compounds for leishmaniasis treatment (Fonseca-Silva et al., 2011; Inacio et al., 2012; Fonseca-Silva et al., 2013; Inacio et al., 2013; Ndjonka et al., 2013; Inacio et al., 2014; Fonseca-Silva et al., 2015; Fonseca-Silva et al., 2016; Emiliano and Almeida-Amaral, 2018; Gervazoni et al., 2018; Inacio et al., 2019; Gervazoni et al., 2020). Apigenin has demonstrated a key role in the treatment of some health issues, such as diabetes, amnesia and Alzheimer’s disease, depression, insomnia, and cancer (Salehi et al., 2019). Moreover, the activity of apigenin has been described against *L. donovani*, *T. brucei rhodesiense*, *T. cruzi*, *Encephalitozoon intestinalis* and *Cryptosporidium parvum* (Mead and McNair, 2006; Tasdemir et al., 2006).

Cellular proliferation of the promastigote forms of *L. infantum* in the presence of apigenin was inhibited in a concentration-dependent manner, presenting an IC_50_ of 29.9 µM. A similar effect of apigenin was observed in the promastigote forms of *L. amazonensis*, which showed an IC_50_ value of 23.7 µM (Fonseca-Silva et al., 2015).

Some conditions for the eligibility of a new drug for the treatment of visceral leishmaniasis have been described. The new drug should have an IC_50_ less than 10 μM against intracellular amastigotes; be, at a minimum, tenfold more active against intracellular amastigotes than against mammalian cells (SI ≥ 10); be active *in vivo*; and cause a reduction in liver parasite load more than 70% in a relevant small animal model after at most 5 doses at 50 mg/kg, preferably when administered by the oral route once or twice per day (Pink et al., 2005; Katsuno et al., 2015).

In the present study, we demonstrated that apigenin was active against intracellular amastigotes, with an IC_50_ of 2.3 µM and a selectivity index of 34.3. We also observed that the treatment of BALB/c mice infected with *L. infantum* with apigenin (1 mg/kg twice a day, reaching a maximum daily dose of 2 mg/kg/day, orally administered) was capable of reducing the liver parasite load by greater than 70% in both therapeutic schemes (short-term and long-term). It is important to point out that the treatment of apigenin did not compromise the overall health of the infected mice, satisfying all the criteria described above. A similar effect was observed with (-)-epigallocatechin 3-*O*-gallate, an abundant flavonoid constituent of green tea, that had an IC_50_ value of 2.6 µM for intracellular amastigotes and demonstrated a reduction of the liver parasite load, reaching 98.7% inhibition at 50 mg/kg/day (Inacio et al., 2019).

Taken together, the results indicate apigenin as a new compound for the treatment of visceral leishmaniasis and support further studies to determine the ideal therapeutic and optimal drug dose regimen.

## Data availability statement

The original contributions presented in the study are included in the article/**Supplementary Material**. Further inquiries can be directed to the corresponding author.

## Ethics statement

The animal study was reviewed and approved by L-11/2017 A2.

## Author contributions

Conceptualization, YE and EA-A; Methodology, YE and EA-A; Validation, YE and EA-A; Formal analysis, YE; Investigation, YE and EA-A; Data curation, YE and EA-A; Writing—original draft preparation, YE and EA-A; Writing—review and editing, EA-A; Supervision, EA-A. All authors contributed to the article and approved the submitted version.
