# Supplementary material for: Apigenin is a promising molecule for treatment of visceral leishmaniasis
Source: Front Cell Infect Microbiol. 2023 Apr 5;13:1066407. doi: 10.3389/fcimb.2023.1066407 (PMC10113494; doi:10.3389/fcimb.2023.1066407)
Supplement: Supplementary file 1 [file DataSheet_1.pdf]

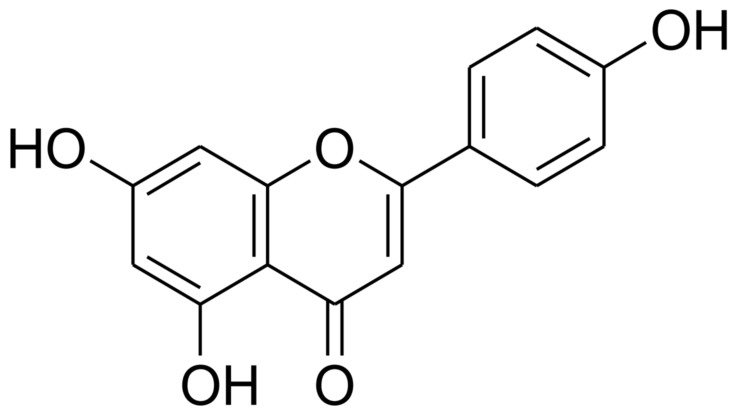


Supplementary Figure 1 - Chemical structures of Apigenin

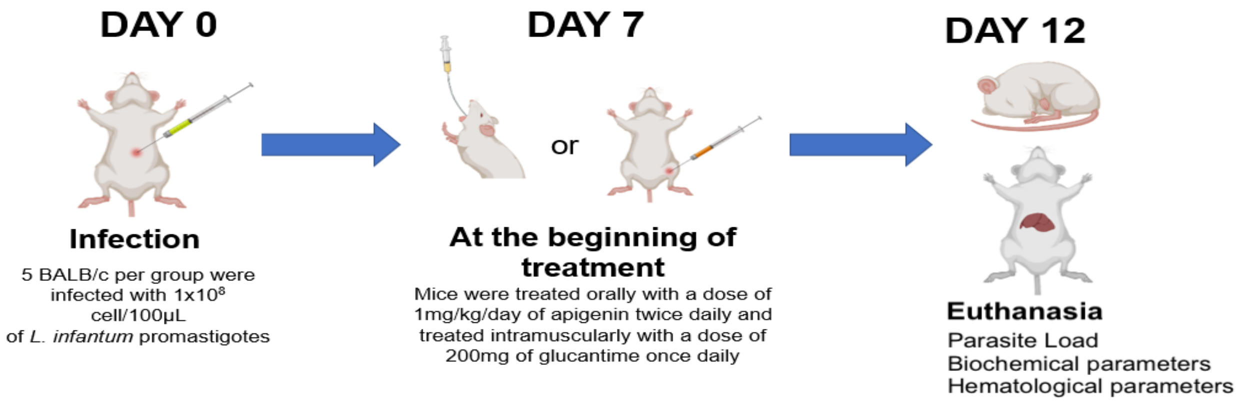


Supplementary Figure 2 – Short-term therapeutic scheme

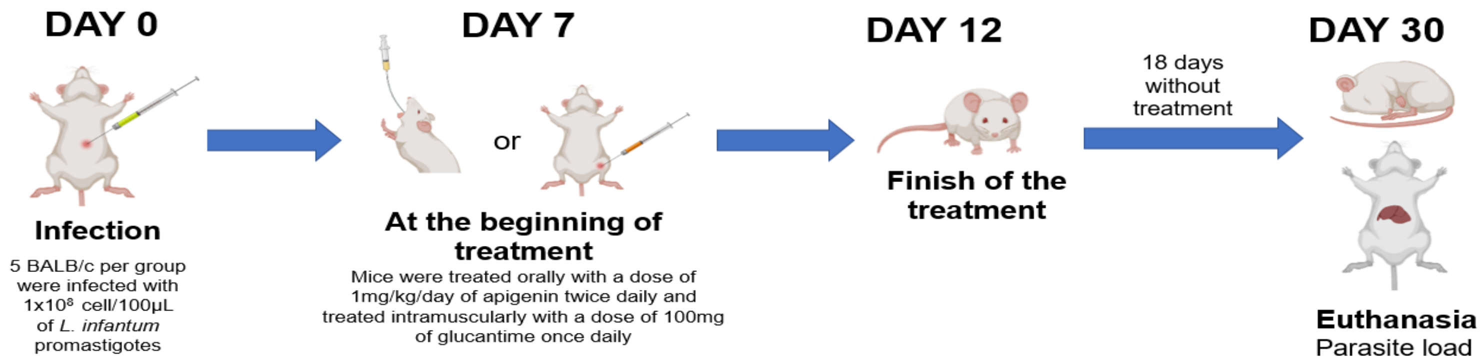


Supplementary Figure 3 – Long-term therapeutic scheme

**Table 1: Biochemical parameters**

| | Reference values | Control | Apigenin | Meglumine antimoniate |
|---|---|---|---|---|
| **Sodium (mEq/L)** | 127-174 | 128 ± 0.83 | 129 ± 0.80 | 127 ± 0.91 |
| **Potassium (mEq/L)** | 4.6 - 8 | 2.5 ± 0.06 | 2.6 ± 0.04 | 5.1 ± 2.08 |
| **Urea (mg/dL)** | 18 - 29 | 38.2 ± 1.40 | 36.1 ± 2.50 | 39.6 ± 0.63 |
| **Albumin (g/dL)** | 2.5 - 4.8 | 2.0 ± 0.04 | 1.8 ± 0.05 | 2.0 ± 0.04 |
| **Calcium (mg/dL)** | 5.9 – 9.4 | 8.3 ± 0.08 | 7.8 ± 0.17 | 8.0 ± 0.05 |
| **Iron (mg/dL)** | 130 - 134 | 214.2 ± 5.65 | 198.4 ± 10.60 | 189.5 ± 9.82 |
| **AST (U/L)** | 59 - 247 | 121 ± 13.59 | 151 ± 28.23 | 127 ± 36.63 |
| **ALT (U/L)** | 28 - 132 | 48.4 ± 2.01 | 63.2 ± 5.00 | 54 ± 8.43 |
| **Creatine Kinase (U/L)** | 68 - 1070 | 133 ± 35.83 | 208 ± 66.75 | 548 ± 167.90 |
| **Alkaline phosphatase (U/L)** | 62 - 209 | 109 ± 8.70 | 108 ± 5.18 | 93.3 ± 5.15 |
| **Cholesterol (mg/dL)** | 36 - 96 | 65 ± 3.50 | 59.6 ± 2.29 | 66.2 ± 3.67 |
| **Total Proteins (g/dL)** | 3.6 – 6.6 | 4 ± 0.08 | 3.9 ± 0.07 | 4 ± 0.04 |

After 5 days of treatment, BALB/c mice were anesthetized, blood was collected, and serum was separated for analysis of biochemical markers. AST = aspartate aminotransferase; ALT = alanine aminotransferase. The values are represented by the mean ± standard error of the experiment with 5 animals in each group. Biochemical parameters were measured by the clinical analysis platform of Instituto de Ciência e Tecnologia em Biomodelos (FIOCRUZ. BR).

**Table 2: Hematological parameters**

| | Reference values | Control | Apigenin | Meglumine antimoniate |
|---|---|---|---|---|
| **RBC (mil/mm3)** | 8.2 – 10.2 | 9.4 ± 0.09 | 9.3 ±0.12 | 9.4 ± 0.18 |
| **Hemoglobin (g/dL)** | 14.4 – 15.7 | 13.8 ± 0.20 | 13.9 ± 0.19 | 13.8 ± 0.29 |
| **Hematocrit (%)** | 59.4 – 49.18 | 47.0 ± 0.56 | 46.6 ± 0.55 | 47.2 ± 1.23 |
| **MCV ($fm^3$)** | 41.3 – 55.5 | 49.7 ± 0.18 | 50 ± 0.15 | 50.5 ± 0.35 |
| **MCH (pg)** | 14.9 – 16.5 | 14.6 ± 0.10 | 14.9 ± 0.09 | 14.7 ± 0.03 |
| **MCHC((g/dL)** | 27.4 - 32 | 29.3 ± 0.10 | 29.7 ± 0.21 | 29.3 ± 0.22 |

After 5 days of treatment. BALB/c mice were anesthetized. blood was collected. and serum was separated for analysis of hematological parameters. RBC = Red Blood Cells. MCV= Mean Corpuscular Volume; MHC = Mean Corpuscular Hemoglobin; MCHC = Mean Corpuscular Hemoglobin Concentration. The values are represented by the mean ± standard error of the experiment with 5 animals in each group. Hematological parameters were measured by the clinical analysis platform of Instituto de Ciência e Tecnologia em Biomodelos (FIOCRUZ. BR).
